# Respiratory Syncytial Virus: An Uncommon Cause of Febrile Seizures—Results from a Systematic Review and Meta-Analysis

**DOI:** 10.3390/pediatric14040055

**Published:** 2022-11-02

**Authors:** Matteo Riccò, Milena Pia Cerviere, Silvia Corrado, Silvia Ranzieri, Federico Marchesi

**Affiliations:** 1 AUSL–IRCCS di Reggio Emilia, Servizio di Prevenzione e Sicurezza Negli Ambienti di Lavoro (SPSAL), Local Health Unit of Reggio Emilia, 42122 Reggio Emilia, Italy; 2 Dipartimento Universitario di Medicina e Chirurgia Traslazionale, Università Cattolica del Sacro Cuore, 00168 Rome, Italy; 3 Department of Medicine DAME—Division of Pediatrics, University of Udine, 33100 Udine, Italy; 4 Department of Medicine and Surgery, University of Parma, 43126 Parma, Italy

**Keywords:** respiratory syncytial virus, respiratory tract infections, seasonal influenza, febrile seizures, epidemiology

## Abstract

Human Respiratory Syncytial Virus (RSV) is a highly contagious viral pathogen. In infants, it is usually listed among the main causes of medical referrals and hospitalizations, particularly among newborns, and a considerable base of evidence associates RSV infections and bronchiolitis with long-term neurological sequelae. We specifically performed a systematic review and meta-analysis in order to ascertain whether RSV infections may be associated with an increased risk for febrile seizures (FS) in infected infants. According to the PRISMA statement, Pubmed, Embase, and pre-print archive medRxiv.og were searched for eligible observational studies published up to 1 July 2022. Raw data included the incidence of FS among children admitted for influenza-like illness (ILI) and/or bronchiolitis, with a confirmed diagnosis of RSV or seasonal influenza virus (SIV) infection. Data were then pooled in a random-effects model. Heterogeneity was assessed using the I^2^ measure, while reporting bias was assessed by means of funnel plots and regression analysis. A total of 11 studies including 6847 cases of RSV infections were retrieved, with a pooled prevalence of 29.975 cases of FS per 1000 RSV cases (I^2^ = 88.5%). The prevalence was not substantially greater in studies performed in pediatric intensive care units (53.817 per 1000 RSV cases vs. 23.552, *p* = 0.12). Higher occurrence of FS was reported from studies performed after 2010 (Risk Ratio [RR] 1.429, 95% Confidence Interval [95%CI] 1.049–1.948), and in China (RR 2.105, 95%CI 1.356–3.266) and South Africa (RR 1.722, 95%CI 1.060–2.824) than in Europe, while a lower occurrence was reported form the USA (RR 0.414, 95%CI 0.265–0.649). Eventually, FS were less likely reported from RSV cases compared to subjects affected by seasonal influenza (RR 0.402; 95%CI 0.228–0.708). Although RSV is often associated with high risk of neurological complications, substantially less cases of FS are reported than in SIV infections. However, the paucity of available studies recommends a cautious appraisal of aforementioned results.

## 1. Introduction

Human Respiratory Syncytial Virus (RSV; genus orthopneumovirus; family Pneumoviridae) is a highly contagious pathogen associated with a high burden of acute, lower respiratory tract infections (LRTI), particularly in infants aged 2 years or less [1,2,3]. It affects even healthy children [4,5], with a well-defined seasonal trend [2,6], causing high rates of hospitalization, irrespective of baseline clinical conditions of the affected individuals [7,8,9,10].

In recent years, a growing amount of evidence has linked RSV infections with an increased risk of chronic conditions in adulthood, including neurologic complications [11]. More precisely, a recent summary of the literature suggests that 1% to 7% of all children hospitalized with RSV infection will experience some sort of both short- and long-term neurologic complications (i.e., childhood encephalitis/encephalopathy; complex seizures, status epilepticus) [11]. These figures represent a substantial burden, as RSV annually causes up to 3.2 million hospitalizations [1,6,12,13,14], and direct costs have to be pooled to the indirect ones associated with the long-term management of RSV infections [15]. When dealing with clinical features, with a reporting rate approaching 85% of cases, seizures have been identified as the most frequent ones, reflecting the heterogeneous nature of the RSV effects on the central nervous system (CNS), similarly to other respiratory viruses such as influenza virus adenovirus, and even SARS-CoV-2 [11,16,17,18,19].

Several CNS infectious (i.e., meningitis, encephalitis, brain abscesses) of viral, bacterial and even fungine etiology are characterized by the occurrence of fever and seizures [20,21,22]. Seizures associated with CNS infections are well distinct from febrile seizures (FS), the latter being the most common seizures or convulsions in young children [20]. FS can be defined as seizures accompanied by a fever of at least 38 °C (100.4 °F) without signs of central nervous system infections. FS commonly occur in children 6 up to 60 months of age [20,21,22], peaking between 12 and 18 months of age [21,22]. Their incidence is estimated between 2% and 5% in Western countries and may peak to 10% in certain Asian populations [20], with a distinctive genetic pattern suggested by the familiar clustering of incident FS cases [21,22]. The majority of reported cases have been linked with either bacterial or viral respiratory tract infections, including seasonal influenza virus, adenovirus, parainfluenza virus, herpesvirus-6, while very little is known about the occurrence of FS during RSV infections [20,23,24]. The working classification of FS has been recently revised through the implementation of the term “fever-associated seizures or epilepsy” (FASE) [21,23,24,25], i.e., a clinical condition where seizures or epilepsy are accompanied by fever. In turn, FASE are classified in (a) simple (i.e., generalized seizures lasting <15 min and not recurring in 24 h), complex (i.e., seizures having focal onset, duration >15 min, recurring in 24 h, associated with pre-existing neurologic deficits, developmental delays, or post-ictal neurologic abnormalities), and prolonged (i.e., febrile status epilepticus) FS, (b) febrile seizures plus (FS+), (c) severe myoclonic epilepsy in the infancy or Dravet’s syndrome; (d) genetic epilepsy with febrile seizures plus (GEFS+), (e) febrile infection-related epilepsy syndrome (FIRES), an abrupt onset refractory status epilepticus usually preceded by a non-specific febrile illness without evidence of CNS infection [20,21,22,25]. Even though the appropriate managing of FS is still debated [23,24], achieving an appropriate and correct diagnosis of the underlying clinical status may contribute to preventing further consequences and limiting potential sequelae, radically improving the eventual prognosis of the affected child [21].

A systematic review synthetizing available reports can therefore be particularly useful to healthcare professionals potentially involved in the managing of RSV infections either in hospital or community settings, improving their understanding of incident cases of FS. As a consequence, a systematic review with meta-analysis was undertaken in order to ascertain whether RSV infections may be associated with an increased risk for febrile seizures (FS) in infected infants compared to other respiratory disorders.

## 2. Materials and Methods

The present systematic review and meta-analysis was performed according to the “Preferred Reporting Items for Systematic Reviews and Meta-Analysis” (PRISMA) guidelines [26]. Research concepts were defined according to the “PICO” strategy (Patient/Population/Problem; Intervention; Control/Comparator; Outcome), as shown in Table 1. More precisely, we included studies performed in small children (aged < 60 months) affected by RSV infections with a diagnosis of FS. As a control group, where available, we retained children affected by RSV infections without FS and children affected by other respiratory disorders (e.g., seasonal influenza) with and without FS.

The review was registered in PROSPERO with the progressive number CRD42022345503.

Two scholarly databases (i.e., PubMed/MEDLINE and EMBASE) and the pre-print server medrxiv.org were searched for relevant studies published up to 1 September 2022. No backward chronological restrictions were applied. The search strategy resulted from the combination of the following keywords (free text and Medical Subject Heading [MeSH] terms, where appropriate): (“seizure*” OR “febrile seizure*” OR “convulsion*”) AND (“RSV” OR “respiratory syncytial virus” OR “bronchiolitis” OR “respiratory virus” OR “virus”). All original research articles available online or through inter-library loan were considered eligible for review if written in any language spoken by the investigators (i.e., Italian, English, German, French, Spanish or Portuguese).

Title and abstract screening were performed through a references management software (Mendeley Desktop Version 1.19.5, Mendeley Ltd., London, UK, 2019) by two independent authors (S.R. and M.P.C.) in order to check their consistence with inclusion criteria. Retrieved studies were included in the analyses when meeting the following inclusion criteria:

1. Reporting on original results: review articles, meta-analyses, case reports, meeting reports and conference abstracts were excluded from both qualitative and quantitative analysis;
2. Diagnosis of RSV infection by means of either polymerase chain reaction or point-of-care tests, while diagnoses based on clinical criteria were excluded from the analyses.
3. Reporting crude number of assessed cases of RSV infections;
4. Reporting a working definition of febrile seizure, which was instrumental to dichotomize cases of febrile seizures from afebrile ones.

As the main outcome of the present study was estimating the occurrence of FS in RSV cases, articles reporting on the occurrence of RSV infections in febrile seizures were excluded from the analyses.

All articles meeting all of the inclusion criteria were retained for the full-text review. The investigators independently read full-text versions of eligible articles. Disagreements were resolved by consensus between the two reviewers; when it was not possible to reach consensus, input from a third investigator (M.R.) was searched and obtained.

Data extracted included:

1. Settings of the study (i.e., timeframe, country, single center vs. multicenter, studies focused pediatric intensive care units [PICU] or not);
2. Number of initially sampled children;
3. Number of RSV cases;
4. Age at diagnosis of RSV;
5. Number of FS episodes;
6. Outcome of FS episodes, and more precisely: whether patients reported any long-term sequelae; whether patients had any electroencephalographic (EEG) anomaly or not; whether patients had any cerebrospinal fluid (CSF) anomaly;
7. Where available, whether the subjects included in the study population had received any previous prophylaxis for RSV through monoclonal antibodies (mAb).

The secondary outcome of the present study was to ascertain whether or not RSV was associated with increased risk for FS compared to Seasonal Influenza Virus (SIV), number of SIV positive individuals among initially sampled children, and the number of FS cases were similarly retained. In analogy to the main outcome, only SIV cases having received a laboratory diagnosis were considered in the main analyses.

After data extraction, the potential risk of bias of retrieved studies was rated through the risk of bias (ROB) tool from the National Toxicology Program (NTP)’s Office of Health Assessment and Translation (OHAT) handbook [27,28]. ROB tool evaluates the internal validity of a given study assessing whether or not the study’s design and/or management have compromised the credibility of the link between exposure and outcome. OHAT ROB tool covers six possible sources of bias (i.e., participant selection, confounding, attrition/exclusion, detection, selective reporting, and other sources), with potential answers ranked from “definitely low”, “probably low”, “probably high”, to “definitely high”, but it does not apply an overall rating for each study. Following OHAT handbook recommendations, even studies with “probably high” or “definitely high” ratings were not removed from quantitative analysis.

Initially, a descriptive analysis was performed by calculating crude prevalence figure per 1000 RSV cases for all retrieved studies, and pooled estimates were then calculated through a random effect model, that was preferred over a fixed effect model in order to cope with the presumptive heterogeneity across the various studies. Risk for FS among RSV cases compared to SIV cases was calculated as a pooled risk ratio (RR), and a random effect model was similarly applied.

The amount of Inconsistency between included studies was estimated by means of I^2^ statistic (i.e., the percentage of total variation across studies that is due to heterogeneity rather than chance), assuming the following categorization: for I^2^ estimates ranging from 0 to 25%, low heterogeneity was assumed; for I^2^ ranging between 26% and 50%, moderate heterogeneity; for I^2^ ≥ 50% the heterogeneity was acknowledged as substantial.

To investigate publication bias, contour-enhanced funnel plots representing Egger test for quantitative publication bias analysis (at a 5% of significance level) were generated. Radial plots were then calculated and visually inspected to rule out small study bias. All analyses were performed by means of “meta” and “metafor” packages with R (version 4.1.1) and RStudio (version 2021.09.0.351) software. The meta package is an open-source add-on for conducting meta-analyses.

## 3. Results

**Figure 1:**
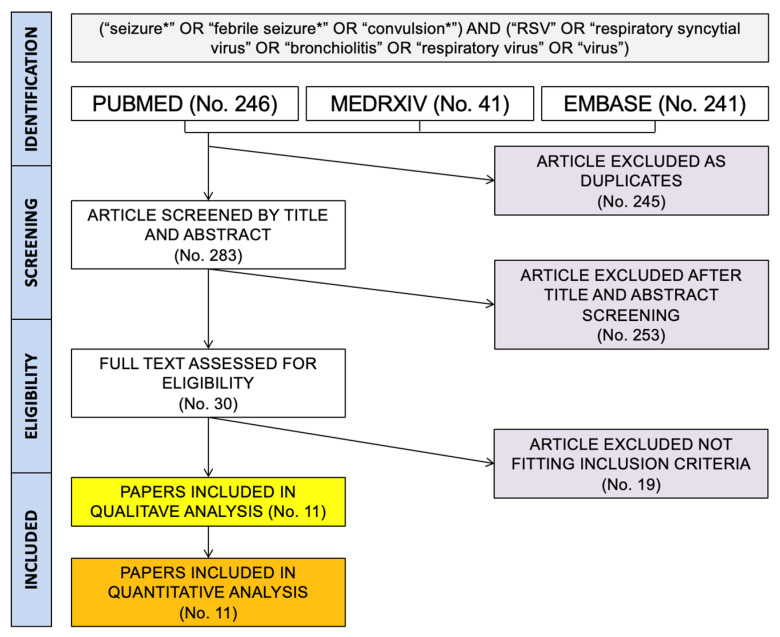
Flow chart of the studies included in the present systematic review and meta-analysis.

As shown in Figure 1, a total pool of 528 entries (of them: 246 from PubMed; 241 from EMBASE, and 96 from medrxiv.org) were initially retrieved. A total of 245 duplicated items were removed, and 283 articles were therefore screened by title and abstract. A total of 253 entries were then removed, while 30 articles were assessed for eligibility, with the subsequent exclusion of 19 items as not fitting inclusion criteria.

Finally, 11 papers were included in qualitative and quantitative analysis, and are summarized in Table 2 and Table 3. Briefly, the studies covered a timeframe ranging from 1993 to 2018, with 7 studies published before 2010 [16,17,29,30,31,32,33], and only 4 further reports since then [34,35,36,37]; 4 of the included studies [16,17,29,30] were from the United States, 2 studies were reports from China or Hong Kong [31,33], while the remaining reports were from Germany [32], South Korea [34], Finland [35], France [36], and South Africa [37]. Most of the aforementioned studies were observational reports from a single center [16,17,29,30,31,33,34,35,36], three of which included cases from PICU [30,33,36], while only two were multicentric reports [32,37].

As shown in Table 2, a total of 26,576 medical records were ultimately retrieved (range: 54 to 7592), including a total of 6874 cases of RSV infections (range: 14 to 1537). Mean age of sampled cases ranged from 1.0 months to 28.1 months, while the actual range was 0.1 months to 43 months. Among the retrieved medical records, 172 (2.6%; range: 0.5% to 10.3%) were complicated by FS.

Of the retrieved studies, a total of four included estimates for SIV diagnosis in the very same parent population [31,33,35,37], for a total of 1183 cases that were paired to 1006 cases of RSV infections, with 231 episodes of FS occurring in SIV cases.

Data on background characteristics of participants were irregularly reported (Table 3). For instance, not only congenital defects or information on miscarriage [16,17,29,30,34,36], electroencephalogram anomalies [16,17,29,30,34,36], and CSF specimens [16,17,29,30,34] were inconsistently reported across the retrieved studies, but data on their actual occurrence in FS episodes associated with RSV infections were also erratically available [30]. Briefly, around 10.1% of sampled episodes occurred in children affected by any birth issue, 52.4% had any EEG anomaly, while only 1 case out of 41 collected specimens (2.4%) has noticeable CSF anomalies. Moreover, data on previous immunization of included children through mAb were included only in the report from Simon et al. [32], but again we were unable to characterize how many of these individuals did develop FS during RSV. Unfortunately, the occurrence of the aforementioned factors in RSV cases not affected by FS episodes was not consistently reported across the retrieved studies.

Quality assessment of retrieved studies is summarized in Table 4. As shown, the majority of samples had either a probably low [17,29,30,31,34,37] or a definitively low risk of selection bias [32,33,35,36], while only the study of Sweetman et al. [16] was presumptively affected by a significant risk of bias because of the relatively unclear selection procedures that led to the identification of the 12 patients included in the report. Conversely, only 5 out 11 reports did account for potential modifying factors in the study design and reporting data [32,33,35,36,37], while 7 of 11 studies clearly included outcome data on reported medical records [17,32,33,34,35,36,37]. Focusing on the detection bias (i.e., systematic differences between groups in how outcomes are determined), the exposure characterization was well defined in all of the reported studies, and the risk of bias in outcome assessment and internal validity were either probably low or definitively low in all reports. On the contrary, the risk for selective reporting bias (i.e., the potential omission of certain data or complete outcomes) was substantial in the majority of included studies [16,17,29,30,31,33], as not all measured outcomes were clearly and homogenously reported.

**Figure 2:**
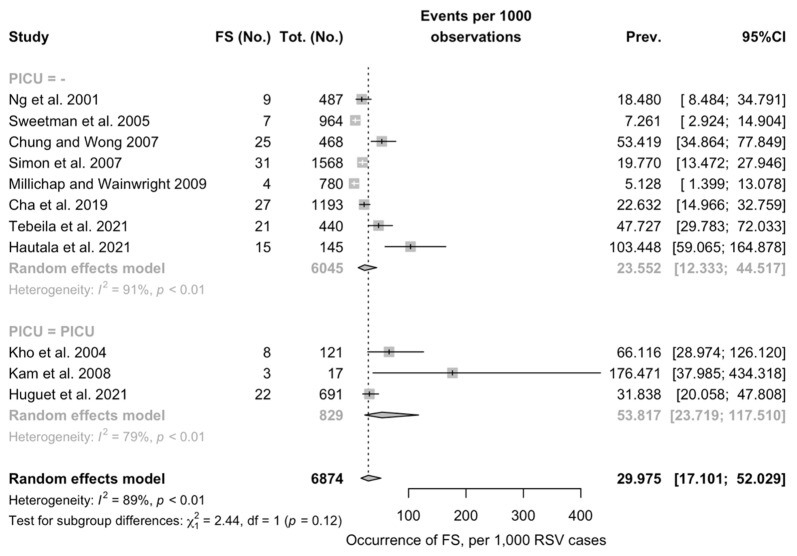
Pooled prevalence of Febrile Seizures (FS) among Respiratory Syncytial Virus (RSV) cases from the retrieved studies. Note: PICU = Pediatric Intensive Care Units; 95%CI = 95% Confidence Intervals; Prev = prevalence [16,17,29,31,32,34,35,37].

Overall, pooled prevalence of FS was estimated in 29.975 cases per 1000 cases of RSV (95%CI 17.101 to 52.029), being not substantially greater among studies including estimates from PICU than in those not including PICU (53.817 per 1000 cases, 95%CI 23.719 to 117.510 vs. 23.552 per 1000 cases, 95%CI 12.333 to 44.517; Chi^2^ = 2.44, *p* = 0.12) (Figure 2). In both sub-analyses, the heterogeneity measured by means of I^2^ statistics was substantial (i.e., 91% for non-PICU studies, 79% for PICU studies, pooled estimate 89%).

As shown in Table 5, a corresponding Risk Ratio (RR) of 1.761 (95%CI 1.181 to 2.585) was identified for FS among patients from PICU than in patients not admitted to PICU. Similarly, an increased occurrence of FS was identified in studied performed in 2010 and in the following years than in those from the previous decades (RR 1.492, 95%CI 1.049 to 1.948). When taking account of the geographic area, and assuming Europe as the reference area, an increased occurrence of FS in RSV cases was associated with China (RR 2.105, 95%CI 1.356 to 3.266), and South Africa (RR 1.722, 95%CI 1.060 to 2.824), while a decreased risk was associated with the USA (RR 0.414, 95%CI 0.265 to 0.659).

**Figure 3:**
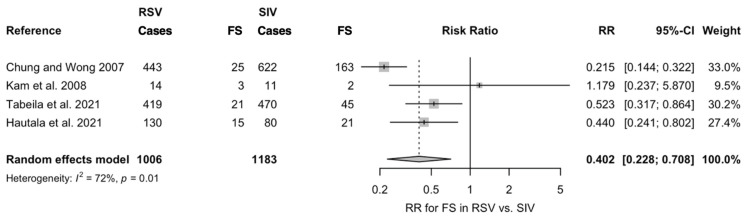
Risk Ratio (RR) for febrile seizures (FS) in cases of Respiratory Syncytial Virus (RSV) infections compared to Seasonal Influenza Virus (SIV) in the four studies that included both laboratory diagnoses (Note: 95%CI = 95% confidence intervals) [31,33,35,37].

As shown in Figure 3, estimates from the four studies that included both SIV and RSV diagnoses led to a pooled incidence of 64 FS episodes out of 1006 RSV infections (6.3%) compared to 231 out of 1183 cases of SIV infections (19.5%). Assuming SIV cases as the reference group, a Risk Ratio (RR) for FS equals to 0.402 (95%CI 0.228 to 0.708) was therefore associated to RSV diagnoses. Again, the I^2^ statistics hinted towards the substantial heterogeneity of retrieved estimates (72%, *p* = 0.01).

**Figure 4:**
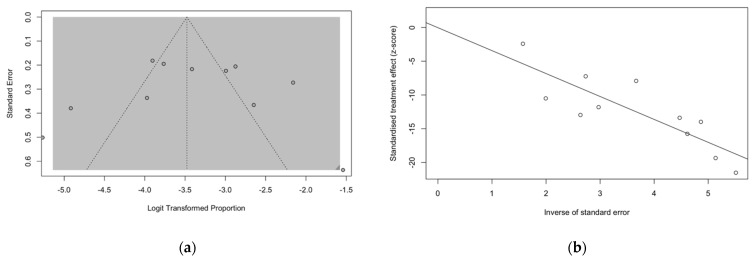
Funnel plots for studies included in the meta-analysis (**a**). Visual inspection suggested that publication could be ruled out, as otherwise confirmed by the results of Egger test (t = −0.01, df = 9, *p*-value = 0.990). (**b**) Similarly, in radial plots (**b**), the estimates for included studies were substantially scattered across the regression line, suggesting the ruling out of a potential small study effect.

Funnel plots and regression tests for funnel plot asymmetry were calculated in order to ascertain the presence of publication bias. In a funnel plot, studies’ effect sizes are plotted against their standard errors. Each point represents a separate study, and their asymmetrical distribution at visual inspection is suggestive of publication bias (i.e., publication depending not just on the quality of the research, but also on the hypothesis tested, and the significance and direction of detected effects), while their scattered distribution suggests that publication bias can be otherwise ruled out, as in Figure 4a. Such subjective evidence from the funnel plot was similarly confirmed after the regression test. In fact, Egger test ruled out publication bias (i.e., t = −0.01, df = 9, *p*-value = 0.990). Similarly in radial plots (Figure 4b), estimates were substantially scattered across the regression line, suggesting no significant small study effect.

## 4. Discussion

Respiratory tract infections are usually associated with an increased risk for FS [20,21,22]; however, while a considerable amount of evidence has linked FS with viral pathogens such as SIV or adenoviruses [21,22,23], their occurrence during RSV infections has remained largely undefined [17].

In our systematic review and meta-analysis, we were able to retrieve a total of 11 studies dealing with FS in RSV infections [16,17,29,30,31,32,33,34,35,36,37], with a pooled occurrence of around 30 episodes per 1000 acute RSV cases. The risk of FS was greater in patients from PICU than in non-PICU centers (RR 1.761; 95%CI 1.181 to 2.585), in studies performed after 2009 (RR 1.492, 95%CI 1.049 to 1.948), that is immediately after the publication of the most recent recommendations for the management of FS [23,24]. Moreover, studies performed China (RR 2.105, 95%CI 1.356 to 3.266) and South Africa (RR 1.722, 95%CI 1.060 to 2.824) were associated with a substantially higher risk for FS than in European based ones, with a conversely decreased risk for those based in the USA (RR 0.414, 95%CI 0.265 to 0.659). Interestingly enough, as 4 studies provided estimates for FS in RSV cases as well as in episodes of SIV infections [31,33,35,37], a direct comparison was performed, and RSV infections were ultimately associated with a substantially reduced risk for FS compared to SIV (RR 0.402, 95%CI 0.228 to 0.708).

In other words, FS in cases of RSV infections do occur, but healthcare providers could expect a substantially lower rate than in other respiratory infections, and particularly among SIV cases. Even though RSV infections have been linked to substantial CNS short- and long-term sequelae [38,39,40], the underlying mechanisms are reasonably quite distinctive from the causes of FS in respiratory disorders, including RSV [38]. While the sequelae of RSV infection have been tentatively associated with direct infection of cells within the CNS [38,41], FS in respiratory viral infection are supposedly the consequence of the “cytokine storm” elicited by the pathogen in the respiratory tract and reaching the CNS [20,21,22,42]. According to some models, the passage of cytokines into the CNS would induce seizures either directly, through their effect on certain neuronal receptors (mostly, GABA receptors), or indirectly, as a consequence of the high temperatures induced by the hypothalamic stimulation, with increased recycling of synaptic vesicles, their enlargement, and the eventual enhancement of synaptic transmission [20,21,22,24,25,39,40,41,43]. Some reports have specifically targeted interleukin (IL) 6 and IL-8 among the key player of the encephalopathy associated with respiratory tract virus infections, including SIV and RSV [39,44,45], that would ultimately lead to the insurgence of FS. While RSV-induced IL-6 and IL-8 production usually requires CNS cells infection, SIV appears quite effective in inducing high levels of these cytokines without the direct invasion of the nervous system [44,45]. In other words, only a reduced subset of RSV cases, i.e., those characterized by more extensive and/or a certain and direct involvement of CNS, would develop noticeable signs of encephalopathy, including FS, irrespective of the background characteristics of the febrile syndrome (e.g., the peak temperature, and the time requested to reach it) [23,38,44]. The higher occurrence of FS among studies including PICU cases may be explained accordingly, i.e., through the higher occurrence of complicated cases, with an invasive RSV infection compared to other clinical reports. Still, this hypothesis is somehow inconsistent with our data. First of all, only a limited share of retrieved studies did include exhaustive data on EEG [16,17,29,30,34,36] and CSF characteristics of FS cases [16,17,29,30,34], urging a very cautious interpretation of summarized data because of the reasonably high selection bias. Second, the share of cases affected by substantial EEG anomalies was relatively low, accounting for 52.4% of assessed episodes. In other words, around half of FS in RSV cases did occur in subjects without any sign of ongoing encephalopathy. Third, only 1 episode of FS was clearly associated with noticeable anomalies within the CSF [29]. In other words, suggestive as it may appear, the association between neuroinvasive RSV infection and FS episodes will require more extensive and appropriately designed studies in order to be eventually confirmed. In this regard, future studies should guarantee a more accurate reporting on two substantial issues represented by risk factors for severe and complicated RSV infections, including [46,47,48]: infants either born at ≤35 weeks of Gestational Age (wGA); children < 2 years of age with chronic lung disease of prematurity (CLD) or hemodynamically significant congenital heart disease (CHD). Moreover, while new preventive options for RSV infections are increasingly made available [49,50,51,52,53], including extended half-life recombinant mAb (e.g., nirsevimab), future studies should more accurately highlight whether or not FS may represent a proxy for treatment failures. As most of RSV cases usually occur at community level, failing to obtain a proper microbiological diagnosis [3,54], if further studies will confirm the association between FS and a more invasive RSV infection pattern, identifying incident cases of FS among children having received previous prophylaxis would help to properly characterize a subset of treated individuals who did not benefit from the delivery of mAb, requiring improved preventive and clinical interventions [50,55,56,57,58,59]. Conversely, the identification of any preventive effect of mAb on FS even in cases of treatment failures would urge for a more extensive definition of medical costs associated with mAb prophylaxis.

*Limits*. Despite the potential interest for healthcare providers involved in the management of FS and RSV cases, our study is affected by several limitations that should be accurately addressed.

First of all, the retrieved articles were of mixed quality, and quite heterogenous in terms of design and overall size. Even though the estimates were reasonably free from small study effects and publication bias, it should be stressed that RSV has been often and improperly regarded as a somewhat “minor” pathogen, and also medical professionals still fail to understand the severity of RSV infections in infants and adults [60,61,62]. Therefore, the large majority of incident cases simply remains undiagnosed [5,52,53,63]. As a consequence, we cannot rule out that our estimates might have been affected by a resulting overestimation, particularly in studies based on records from PICU, where a higher share of complicated cases of RSV infections are reasonably included [16,29,33]. This sampling issue may be substantial in certain studies where a community-based cohort was assessed for RSV infection only in case of hospital admission, a study design that reasonably excluded from the overall estimates milder cases of RSV infection [37]. Not coincidentally, when the occurrence in studies based on PICU were compared with those from non-PICU records, the former scored a noticeably increased incidence rate (53,817 per 1000 cases vs. 23,552). The significance of the sampling issue is also stressed by the heterogenous occurrence of FS among sampled studies when assessed by their geographical settings. Despite some genetic risk factors for FS during respiratory infections being reported [20,21,22,38,39,44], including a substantially higher risk for FS in individual of Asiatic descent [20,22,35], we cannot rule out that our data more simply did reflect a different approach to hospitalization for respiratory infectious syndromes, with higher rates reflecting a more selective approach to hospitalizations.

Second, all the reported studies were performed well before the inception of the SARS-CoV-2 pandemic. Even though RSV and SARS-CoV-2 are clearly unrelated pathogens, the implementation and the subsequent lifting of non-pharmaceutical interventions (NPI; i.e., public health measures that aim to prevent and/or control SARS-CoV-2 transmission in the community) have dramatically influenced the seasonality of RSV infections, with their substantial disappearance during the first half of 2020, when lockdown measures were extensively implemented [64,65,66,67,68], impairing the normal transmission of RSV to susceptible individuals at the community level [66,67,69,70,71,72,73]. Still, RSV has not simply disappeared, and the reduced circulation during the first half of 2020 has generated a large RSV-vulnerable population [53,69,70,71,74], that has been affected by a reemergent RSV epidemics in 2021–2022 [5,52,70,71,74,75]. In other words, summarized data may be hardly comparable to the current real-world experience of most of healthcare providers involved in the managing of infants, RSV cases and/or FS.

Third, even the comparisons with the occurrence of FS in SIV should be cautiously assessed. On the one hand, SIV infections represent a very common condition and, likewise RSV, the large majority of incident cases remains undiagnosed and managed at community level, with only more severe and complicated cases requiring hospitalization [76,77]. On the other hand, the association between FS and SIV infection is well consolidated by solid evidence [20,42,78]. As our study only retrieved a total of 1183 SIV infections with 231 episodes of FS, that were compared to a similarly limited amount of FS episodes in RSV cases (i.e., 64 over 1006 infections), resulting estimates are hardly generalizable, not only in terms of incidence estimates, but also in term or resulting risk estimates. With a potential oversampling of complicated and more severe cases of SIV infection, the seemly reduced share of FS episodes in RSV cases compared to those occurring in individuals affected by seasonal influenza may simply reflect a more selective approach towards hospitalization for SIV cases.

## 5. Conclusions

In summary, our study seemingly does not confirm a strong responsibility of the RSV in causing FS among affected children, as FS represented a relatively uncommon occurrence among RSV cases. However, as coexisting risk factors were inconsistently reported among retrieved studies, and because of the inconsistent testing strategies, it is reasonable that a substantial share of RSV cases may have been lost from the parent estimates, eventually impairing the reliability of our estimates through the oversampling of more complicated cases. As a consequence, our results could hardly be considered definitive, rather urging for additional studies. Through a more consistent case definition, a more extensive retrieval of medical records, and well-defined sampling strategy, a better identification of the true burden of FS in RSV infections would contribute to a more accurate definition of preventive and clinical strategies.

## Figures and Tables

**Table 1:** PICO worksheet (note: RSV = respiratory syncytial virus).

Item	Definition
Population of interest	Children (age < 5 years) affected by RSV infection
Investigated result	Febrile Seizures (FS)
Control	Children affected by RSV infection without FS Children affected by other respiratory virus infections with and without FS
Outcome	Occurrence of FS in RSV infected children

**Table 2:** Summary of included studies.

Reference	Country	Timeframe (Year)	Settings	Description	Mean Age (SD or Range) (Months)	Total Population (No.)	RSV Cases (No./TOT, %)	FS Cases in RSV (No./RSV, %)
Ng et al. [29]	USA (Texas)	1994–1998	SC	All children admitted to the parent centre with bronchiolitis and RSV positive testing; seizure associated with encephalopathy.	8.2 (7.6)	487	487, 100%	9, 1.9%
Kho et al. [30]	USA (Texas)	1996–1998	SC, PICU	Pediatric ICU, consecutive patients admitted with bronchiolitis, viral pneumonitis, upper respiratory tract infections.	5.2 (0.25–22)	4861	121, 2.5%	8, 6.6%
Sweetman et al. [16]	USA (New Mexico)	1993–2003	SC	All children admitted to the parent centre with bronchiolitis and RSV positive testing	7.9 (6.6)	964	964, 100%	7, 0.7%
Chung and Wong [31]	China (Hong Kong)	1998–2003	SC	Retrospective analysis of medical records of patients with a diagnosis of FS.	25.2 (13.2)	923	468, 50.7%	25, 5.3%
Simon et al. [32]	Germany	1999–2005	MC	All inpatients treated for at least 24 h with a virologically confirmed RSV infection irrespective of age, underlying illness, and other comorbidities (RSV season; November–April); 14 pediatric centres	5.3 (term) 4.7 (pre-term) (23–43)	1568	1568, 100%	31, 2.0%
Kam et al. [33]	China	2003–2007	SC, PICU	Retrospective study from a single centre PICU.	10.8 (1–25)	54	17, 31.5%	3, 17.6%
Millichap and Wainwright [17]	USA (Chicago)	2005–2008	SC	Retrospective analysis at a single centre; diagnosis of RSV infection at admission	(0.1–36)	780	780, 100%	4, 0.5%
Cha et al. [34]	Korea	2011–2016	SC	Retrospective analysis of medical records of patients with a diagnosis of RSV (single centre)	20.8 (16.6)	1193	1193, 100%	27, 2.3%
Hautala et al. [35]	Finland	2013–2017	SC	Prospective cohort study on the respiratory viral etiology of FS and a case-control study on the febrile response to FS (23,895 total visits)	28.1 (17.4)	7592	145, 1.9%	15, 10.3%
Huguet et al. [36]	France	2010–2018	SC, PICU	Single centre PICU, all consecutive patients admitted with a diagnosis of bronchiolitis	1.0 (0.6–1.7)	1028	691, 67.2%	22, 3.2%
Tebeila et al. [37]	South Africa	1998–2000	MC	Secondary analysis of data derived from a cohort (No. 39,830 children) of children enrolled into a vaccine safety study, with a total of 7126 hospitalizations	25.0 (17.2–34.8)	7126	440, 6.17%	21, 4.8%

Notes: RSV = respiratory syncytial virus; FS = febrile seizures; SC = single center; MC = multicentric study; PICU = pediatric intensive care unit.

**Table 3:** Main characteristics of the febrile seizures (FS) episodes included in the analysis.

Reference	FS Episodes (No.)	Miscarriage/Discharge (No., %)	EEG Anomalies (No., %)	CSF Anomalies (No., %)
Ng et al. [29]	9	0, -	5, 55.6%	1, 11.1%
Sweetman et al. [16]	7	1, 14.3%	3, 42.9%	0, -
Chung and Wong [31]	-	-	-	-
Simon et al. [32]	-	-	-	-
Kam et al. [33]	-	-	-	-
Millichap and Wainwright [17]	4	0, -	3, 75.0%	0, -
Cha et al. [34]	21	-	6, 28.6%	0, -
Hautala et al. [35]	27	0, -	-	-
Huguet et al. [36]	22	6, 27.3%	16, 72.7%	-
Tebeila et al. [37]	-	-	-	-
Pooled		7/69 10.1%	33/63 52.4%	1/41 2.4%

Note: EEG = electroencephalogram, CSF = cerebrospinal fluid.

**Table 4:** Summary of risk of bias assessment according to the risk of bias (ROB) tool from the National Toxicology Program (NTP)’s Office of Health Assessment and Translation (OHAT) handbook [27,28].

	Ng et al. [29]	Kho et al. [30]	Sweetman et al. [16]	Chung and Wong et al. [31]	Simon et al. [32]	Kam et al. [33]	Millichap and Wainwright [17]	Cha et al. [34]	Hautala et al. [35]	Huguet et al. [36]	Tebeila et al. [37]
Selection bias											
Did selection of study participants result in appropriate comparison groups?	PL	PL	PH	PL	DL	DL	PL	PL	DL	DL	PL
Confounding bias											
Did the study design or analysis account for important and modifying variables	PH	PH	PH	PH	PL	PL	PH	PH	DL	DL	PL
Exclusion bias											
Were outcome data complete without attrition or exclusion from analysis?	PH	PH	PH	PH	PL	PL	PL	PL	PL	PL	PL
Detection bias											
Can we be confident in the exposure characterization?	DL	DL	DL	DL	DL	DL	DL	DL	DL	DL	DL
Can we be confident in the outcome assessment?	PL	PL	PL	PL	DL	PL	PL	PL	DL	DL	DL
Selective reporting bias											
Were all measured outcomes reported?	DH	DH	DH	DH	PL	DH	DH	PL	DL	DL	DL
Other sources of bias											
Were there no other potential threats to internal validity (e.g., statistical methods were appropriate, and researchers adhered to the study protocol)?	DL	DL	DL	DL	DL	DL	DL	DL	DL	DL	DL

Note: PL = probably low; PH = probably high; DL = definitively low; DH = definitively high.

**Table 5:** Occurrence of febrile seizures (FS) in the collected studies reporting on cases of Respiratory Syncytial Virus (RSV) infection. Notes: RR = Risk Ratio, 95%CI = 95% Confidence Intervals; PICU = pediatric intensive care unit.

	Total RSV Cases (No./6874, %)	FS Cases (No./RSV Cases, %)	RR (95%CI)	*p* Value
Timeframe				
<2010	4405, 65.7%	87, 2.0%	1.000	Reference
≥2010	2469, 34.3%	85, 3.4%	1.429 (1.049; 1.948)	0.025
Area				
Europe	2404, 35.0%	68, 2.8%	1.000	Reference
USA	2352, 34.2%	28, 1.2%	0.414 (0.265; 0.649)	<0.001
China	485, 7.1%	28, 5.8%	2.105 (1.356; 3.266)	0.001
South Africa	440, 6.4%	21, 4.8%	1.722 (1.060; 2.824)	0.031
South Korea	1193, 17.4%	28, 2.3%	0.796 (0.510; 1.235)	0.319
Settings				
Multicentric	2008, 29.2%	52, 2.6%	1.063 (0.759; 1.483)	0.718
Single Centre	4866, 70.7%	120, 2.5%	1.000	Reference
PICU	829, 12.1%	33, 4.0%	1.761 (1.181; 2.585)	0.004
No PICU	6045, 87.9%	139, 2.3%	1.000	Reference

## Data Availability

Data are available on request to the corresponding Author.
